# Porcine epidemic diarrhea virus: An emerging and re-emerging epizootic swine virus

**DOI:** 10.1186/s12985-015-0421-2

**Published:** 2015-12-22

**Authors:** Changhee Lee

**Affiliations:** Animal Virology Laboratory, School of Life Sciences, BK21 Plus KNU Creative BioResearch Group, Kyungpook National University, Daegu, 41566 Republic of Korea

**Keywords:** PED, PEDV, Review, Molecular epidemiology, Diagnosis, Pathogenesis, Preventive measures

## Abstract

The enteric disease of swine recognized in the early 1970s in Europe was initially described as “epidemic viral diarrhea” and is now termed “porcine epidemic diarrhea (PED)”. The coronavirus referred to as PED virus (PEDV) was determined to be the etiologic agent of this disease in the late 1970s. Since then the disease has been reported in Europe and Asia, but the most severe outbreaks have occurred predominantly in Asian swine-producing countries. Most recently, PED first emerged in early 2013 in the United States that caused high morbidity and mortality associated with PED, remarkably affecting US pig production, and spread further to Canada and Mexico. Soon thereafter, large-scale PED epidemics recurred through the pork industry in South Korea, Japan, and Taiwan. These recent outbreaks and global re-emergence of PED require urgent attention and deeper understanding of PEDV biology and pathogenic mechanisms. This paper highlights the current knowledge of molecular epidemiology, diagnosis, and pathogenesis of PEDV, as well as prevention and control measures against PEDV infection. More information about the virus and the disease is still necessary for the development of effective vaccines and control strategies. It is hoped that this review will stimulate further basic and applied studies and encourage collaboration among producers, researchers, and swine veterinarians to provide answers that improve our understanding of PEDV and PED in an effort to eliminate this economically significant viral disease, which emerged or re-emerged worldwide.

## Background

### Historical perspective

In 1971, British veterinary clinicians noted the appearance of a previously unrecognized enteric disease in growing and fattening pigs [1]. A clinical presentation of watery diarrhea was similar to symptoms of the porcine transmissible gastroenteritis virus (TGEV) infection. However, in the latter case, nursing piglets were only mildly affected. The disease, named epidemic viral diarrhea (EVD), then spread to multiple swine-producing countries in Europe. Five years later, TGE-like EVD re-emerged and in contrast to previous outbreaks, the disease occurred in pigs of all ages including suckling pigs. Therefore, EVD in 1976 was classified as EVD type 2 in order to differentiate it from the initial EVD type 1 condition [2, 3]. In 1978, scientists at the Ghent University in Belgium were the first research group, which partially fulfilled Koch’s postulates and described a coronavirus-like agent (CV777) as the causative pathogen. Furthermore, they provided evidence that this novel virus was distinct from the two known porcine coronaviruses, TGEV and hemaggultinating encephalomyelitits virus [4, 5]. Since then, the EVD disease has been known as “porcine epidemic diarrhea (PED)”.

Since the 1990s, PED cases have become rare in Europe and PED virus (PEDV)-associated diarrhea has been usually observed in adult pigs, whereas suckling piglets were generally spared or developed only mild symptoms [6]. PED was first reported in Asia in 1982 and since then it has had a great economic impact on the Asian pork industry [7–11]. In contrast to the present situation in Europe, PED epizootics in Asia are more severe causing high mortality in neonatal piglets and the disease has become an endemic pattern recently. However, despite a notorious reputation in Asian swine-producing countries, PED was not a well-known disease worldwide. For example, the disease had never occurred in the United States until 2013. In May 2013, PED suddenly appeared in the United States and rapidly spread across the country, as well as to Canada and Mexico, causing deaths of more than 8 million newborn piglets in the United States alone during a 1 year-epidemic period [12–15]. Subsequently, severe PED outbreaks recurred in Asian countries including South Korea, Taiwan, and Japan [16–18]. PEDV has now emerged or re-emerged as one of the most devastating viral diseases of swine in the world, leading to significant financial concerns in the global pork industry.

This paper is a brief review focusing on current understanding of the molecular biology, epidemiology, diagnosis, and pathogenesis of PEDV, as well as control strategies to prevent PEDV infection.

## Review

### The virus

#### PEDV genome and virion structures

**Fig. 1:**
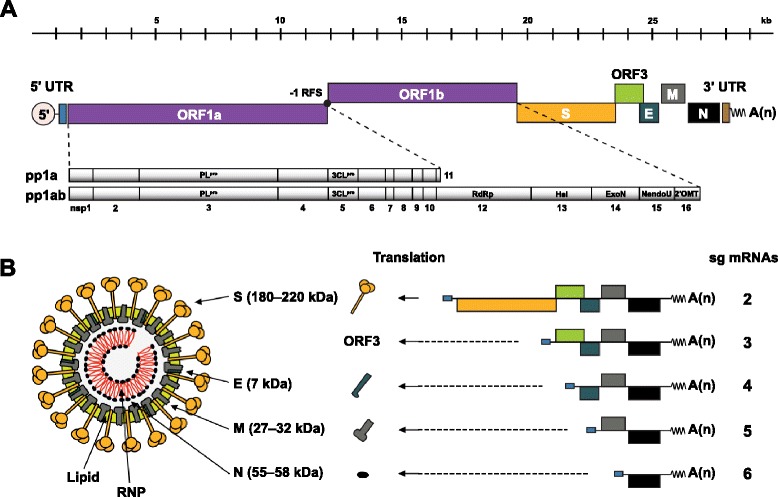
Schematic representations of PEDV genome organization and virion structure. **a** The structure of PEDV genomic RNA. The 5’-capped and 3’-polyadenylated genome of approximately 28 kb is shown at the top. The viral genome is flanked by UTRs and is polycistronic, harboring replicase ORFs 1a and 1b followed by the genes encoding the envelope proteins, the N protein, and the accessory ORF3 protein. S, spike; E, envelope; M, membrane; N, nucleocapsid. Expression of the ORF1a and 1b yields two known polyproteins (pp1a and pp1ab) by −1 programmed RFS, which are co-translationally or post-translationally processed into at least 16 distinct nsps designated nsp1–16 (bottom). PL^pro^, papain-like cysteine protease; 3CL^pro^, the main 3C-like cysteine protease; RdRp; RNA-dependent RNA polymerase; Hel, helicase; ExoN, 3’ → 5’ exonuclease; NendoU, nidovirus uridylate-specific endoribonuclease; 2’OMT, ribose-2’-O-methyltransferase. **b** Model of PEDV structure. The structure of the PEDV virion is illustrated on the left. Inside the virion is the RNA genome associated with the N protein to form a long, helical ribonucleoprotein (RNP) complex. The virus core is enclosed by a lipoprotein envelope, which contains S, E, and M proteins. The predicted molecular sizes of each structural protein are indicated in parentheses. A set of corresponding sg mRNAs (sg mRNA; 2–6), through which canonical structural proteins or nonstructural ORF3 protein are exclusively expressed via a co-terminal discontinuous transcription strategy, are also depicted on the right

PEDV, the etiological agent of PED, is a large-enveloped RNA virus, which is a member of the genus *Alphacoronavirus* within the *Coronaviridae* family placed with the *Arteriviridae* family in the order *Nidovirales* on the basis of similarities in genome organization and replication strategy [4, 6, 19]. The PEDV genome is approximately 28 kb long with a 5’ cap and a 3’ polyadenylated tail and comprises a 5’ untranslated region (UTR), at least 7 open reading frames (ORF1a, ORF1b, and ORF2–6), and a 3’ UTR [20]. The two large ORFs 1a and 1b occupy the 5’-proximal two-thirds of the genome coding for nonstructural proteins (nsps). ORF1a translation yields a replicase polyprotein (pp) la, whereas ORF1b is expressed by a −1 ribosomal frame shift (RFS), which C-terminally extends ppla into pp1ab. These ppla and pplab are post-translationally cleaved by internal proteases generating 16 processing end products, named nsp1–16. The remaining ORFs in the 3’-proximal genome region encode four structural proteins expressed from the respective 3’-co-terminal nested set of subgenomic (sg) mRNAs: the 150–220 kDa glycosylated spike (S) protein, 20–30 kDa membrane (M) protein, 7 kDa envelope (E) protein, 58 kDa nucleocapsid (N) protein, and one accessory gene ORF3 (Fig. 1a) [6, 20–22].

The PEDV genome is encapsulated by a single N protein, forming a long and helical coil structure that is wrapped in a lipid envelope containing 3 surface-associated structural proteins, S, M, and E (Fig. 1b). Enveloped virions are roughly spherical and pleomorphic with a diameter ranging from 95 to 190 nm, including the widely spaced, club-shaped, trimerized S projections measuring 18–23 nm in length [4]. PEDV has a buoyant density of 1.18 g/ml in sucrose and is sensitive to ether and chloroform. The virus is stable at 4–50 °C and is absolutely inactivated at pH values beyond pH 4–9 range [23]. Therefore, PEDV is inactivated by various acidic or alkaline disinfectants [24].

#### PEDV structural proteins

Among viral structural proteins, the S protein of PEDV is the major envelope type I glycoprotein of the virion, which interacts with the cellular receptor during virus entry and stimulates induction of neutralizing antibodies in the natural host [22, 25, 26]. In addition, it is associated with growth adaptation in vitro and attenuation in vivo [27]. Thus, the PEDV S glycoprotein is known to be an appropriate viral gene for determining the genetic relatedness among PEDV isolates and for developing diagnostic assays and effective vaccines [16, 26, 28–30]. The M protein, the most abundant component of the viral envelope, is required for the assembly process and can also elicit the production of protective antibodies with virus-neutralizing activity [31, 32]. The small envelope E protein plays an important role during coronavirus budding, and coexpression of E and M proteins can form spike-less coronavirus-like virions [33]. PEDV E and N proteins are found in the endoplasmic reticulum (ER) where they independently induce ER stress [34, 35]. The N protein has multiple functions in viral replication and pathogenesis in coronavirology [36]. Generally, N proteins of coronaviruses interact with viral genomic RNA and associate with other N protein molecules to protect the viral genome, serving as the critical basis for the helical nucleocapsid during coronavirus assembly [36]. The PEDV N protein also perturbs antiviral responses by antagonizing interferon production, as part of the immune evasion strategy, and activates NF-κB [35, 37]. The product of ORF3, the only accessory gene in PEDV, is thought to function as an ion channel and to influence virus production and virulence [38, 39].

#### PEDV-host interactions

**Fig. 2:**
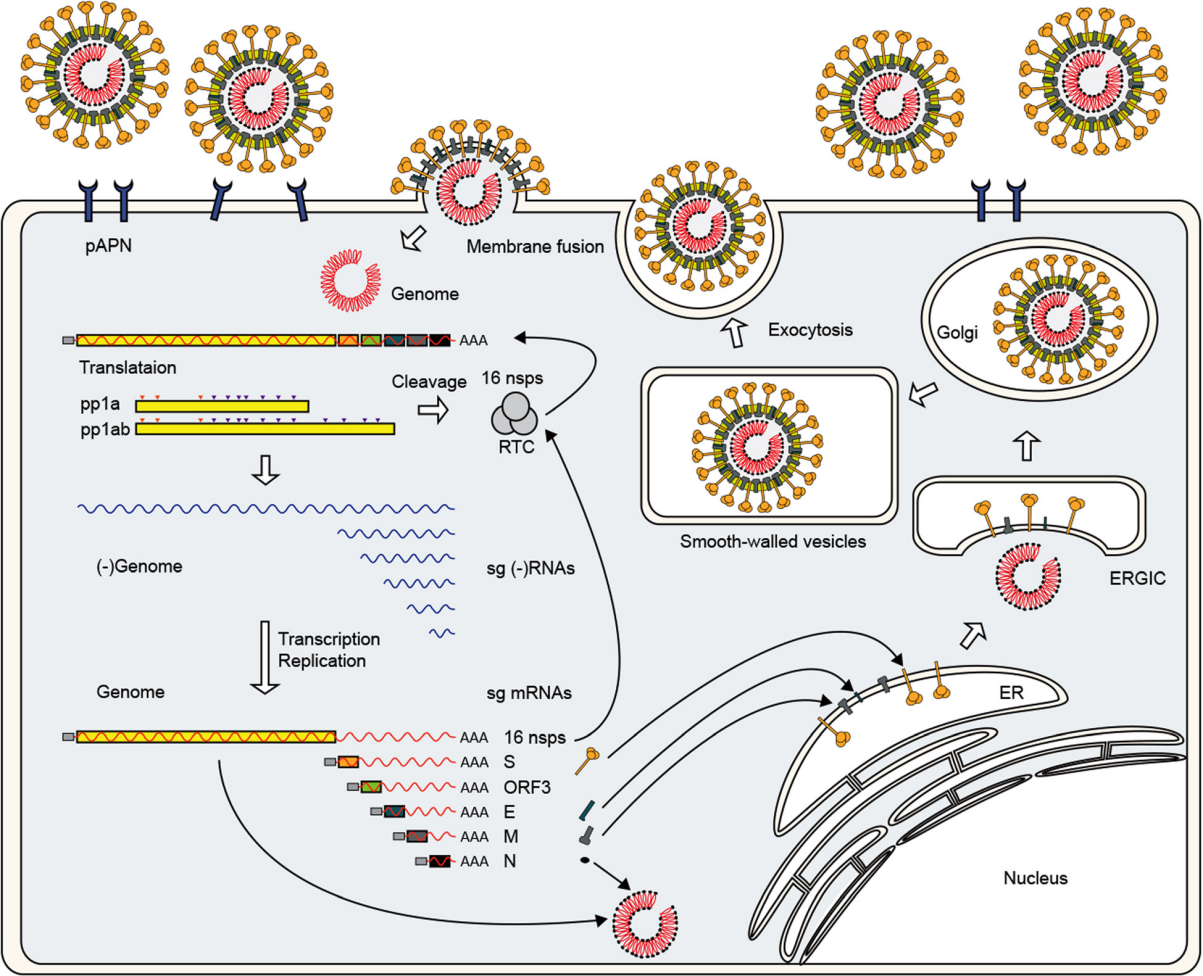
Overview of the PEDV replication cycle. PEDV binds pAPN via the spike protein. Penetration and uncoating occur after the S protein-mediated fusion of the viral envelope with the plasma membrane. Following disassembly, the viral genome is released into the cytoplasm and immediately translated to yield replicases ppla and pp1ab. These polyproteins are proteolytically cleaved into 16 nsps comprising the replication and transcription complex (RTC) that first engages in the minus-strand RNA synthesis using genomic RNA. Both full- and sg-length minus strands are produced and used to synthesize full-length genomic RNA and sg mRNAs. Each sg mRNA is translated to yield only the protein encoded by the 5’-most ORF of the sg mRNA. The envelope S, E, and M proteins are inserted in the ER and anchored in the Golgi apparatus. The N protein interacts with newly synthesized genomic RNA to form helical RNP complexes. The progeny virus is assembled by budding of the preformed RNP at the ER-Golgi intermediate compartment (ERGIC) and then released by the exocytosis-like fusion of smooth-walled, virion-containing vesicles with the plasma membrane [22]

Coronaviruses can infect a wide range of mammals, including humans, bats, and whales, and birds, but typically they have a limited host range, infecting only their specific natural host. Furthermore, coronaviruses exhibit a marked tropism for epithelial cells of the respiratory and enteric tracts, as well as for macrophages [40–42]. PEDV also has a restricted tissue tropism and replicates efficiently in porcine small intestinal villous epithelial cells or enterocytes. Porcine aminopeptidase N (pAPN) predominantly expressed on the surface of epithelial cells of small intestine has been identified as the cellular receptor for PEDV [43, 44]. The N-terminal region of the PEDV spike protein S1 domain is important for recognizing the pAPN receptor [45]. Thus, PEDV entry begins with the binding to pAPN followed by internalization of the virus into target cells by direct membrane fusion, and a subsequent release of the viral genome into the cytosol after uncoating to start the genome replication (Fig. 2). In addition to replicating in primary target cells from the natural host, PEDV can grow in some African green monkey kidney cell lines (Vero and MARC-145) [46, 47]. Although it still remains to be determined whether APN acts as the functional receptor for PEDV on these cells, overexpression of exogenous pAPN renders non-permissive cells susceptible to PEDV infection. This observation suggests a functional significance of the APN receptor density for PEDV propagation in cell culture [44]. A recent study also revealed that cell-surface heparan sulfate acts as the attachment factor of PEDV in Vero cells [48]. The addition of trypsin is indispensable for the isolation and serial passages in Vero cells [28, 46, 49, 50]. Trypsin facilitates PEDV entry and release by cleaving the S protein into S1 and S2 subunits, enabling efficient viral replication and spreading in vitro [51, 52]. However, some cell-adapted attenuated PEDV strains, such as SM98-1 and 83P-5, can support PEDV propagation in the absence of trypsin [44, 53]. As a result of viral infection, distinct cytopathic effects (CPEs) including cell fusion, vacuolation, syncytium, and detachment are produced in infected Vero cells [49].

Since viruses are obligate intracellular parasites, they may adjust the activity of cellular factors or signaling pathways to benefit their own multiplication in host cells. Proteome analysis showed that the expression of proteins involved in apoptosis, signal transduction, and stress responses is affected in PEDV-infected Vero cells [54]. PEDV induces apoptotic cell death in vitro and in vivo through the caspase-independent mitochondrial apoptosis-inducing factor (AIF) pathway [55]. PEDV infection activates the three major mitogen-activated protein kinase (MAPK) cascades involving extracellular signaling-regulated kinase (ERK), p38 MAPK, and c-Jun N-terminal kinase (JNK) [55, 56] (Kim Y, Lee C, unpublished data). In addition, PEDV appears to induce ER stress and activate NF-κB [34, 35]. Therefore, viral replication and subsequent pathological changes rely on PEDV ability to exploit multiple intracellular processes, such as apoptosis, MAPK signaling, and ER stress, which emerge in response to various extracellular stimuli.

#### Heterogeneity

**Fig. 3:**
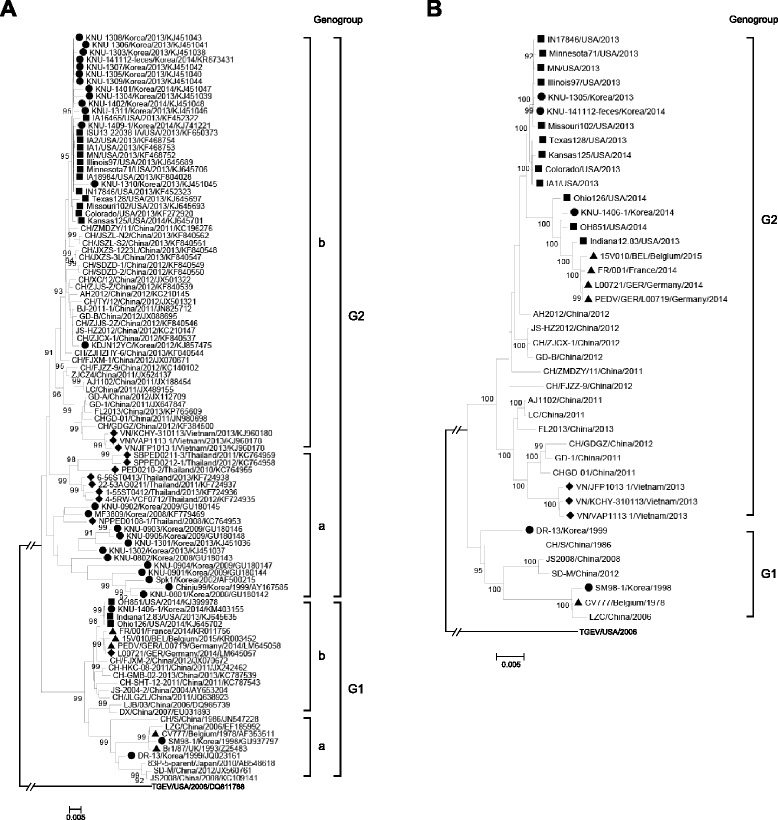
Phylogenetic analyses of global PEDV strains based on nucleotide sequences of the spike genes (**a**) and full-length genomes (**b**). A putative similar region of the spike protein and the complete genome sequence of TGEV was included as an outgroup in each panel. Multiple sequence alignments were performed using ClustalX 2.0 program and the phylogenetic tree was constructed from aligned nucleotide sequences using the distance-based neighbor-joining method of MEGA5.2 software. Numbers at each branch represent bootstrap values greater than 50 % of 1000 replicates. Names of the strains, countries and years of isolation, GenBank accession numbers, genogroups, and subgroups are shown. PEDV isolates identified in different countries are indicated by corresponding symbols: Europe (*solid triangles*), South Korea (*sold circles*), Thailand and Vietnam, (*sold diamonds*), and the United States (*solid squares*). Scale bars indicate nucleotide substitutions per site

Coronaviruses possess the largest known RNA genomes. Nonetheless, they maintain the stability and high fidelity replication of their large genomes while concomitantly generating genetic diversity required for adaptation and emergence. These properties can be ascribed to the 3’-to-5’ proofreading exoribonuclease activity within nsp14 [57, 58]. Considering the high fidelity of coronavirus RNA replication, PEDV is assumed to undergo a slow evolutionary process accumulating mutations or recombination events necessary for viral fitness [59]. Genetic and phylogenetic analyses using the whole-genome or some individual genes have been conducted to determine diversity and relationships of global PEDV isolates. Among these, the full-length S gene and its S1 portion (aa 1–735) have been known to be suitable loci for sequencing to investigate genetic relatedness and molecular epidemiology of PEDV [16, 26, 28, 60, 61]. Although only one serotype of PEDV has been reported, phylogenetic studies of the S gene suggested that PEDV can be genetically separated into 2 groups: genogroup 1 (G1; classical) and genogroup 2 (G2; field epidemic or pandemic). Each genogroup can be further divided into subgroups 1a and 1b, and 2a and 2b, respectively (Fig. 3). G1a includes the prototype PEDV strain CV777, vaccine strains, and other cell culture-adapted strains, whereas G1b comprises new variants that were first identified in China [9], later in the United States [62] and South Korea [61], and recently in European countries [63–65]. G2 contains global field isolates, which are further clustered into 2a and 2b subgroups responsible for previous local epidemic outbreaks in Asia and recent pandemic outbreaks in North America and Asia, respectively. The global outbreak of virulent G2b strains appears to have resulted from point mutations in resident virulent field G1a populations.

**Fig. 4:**
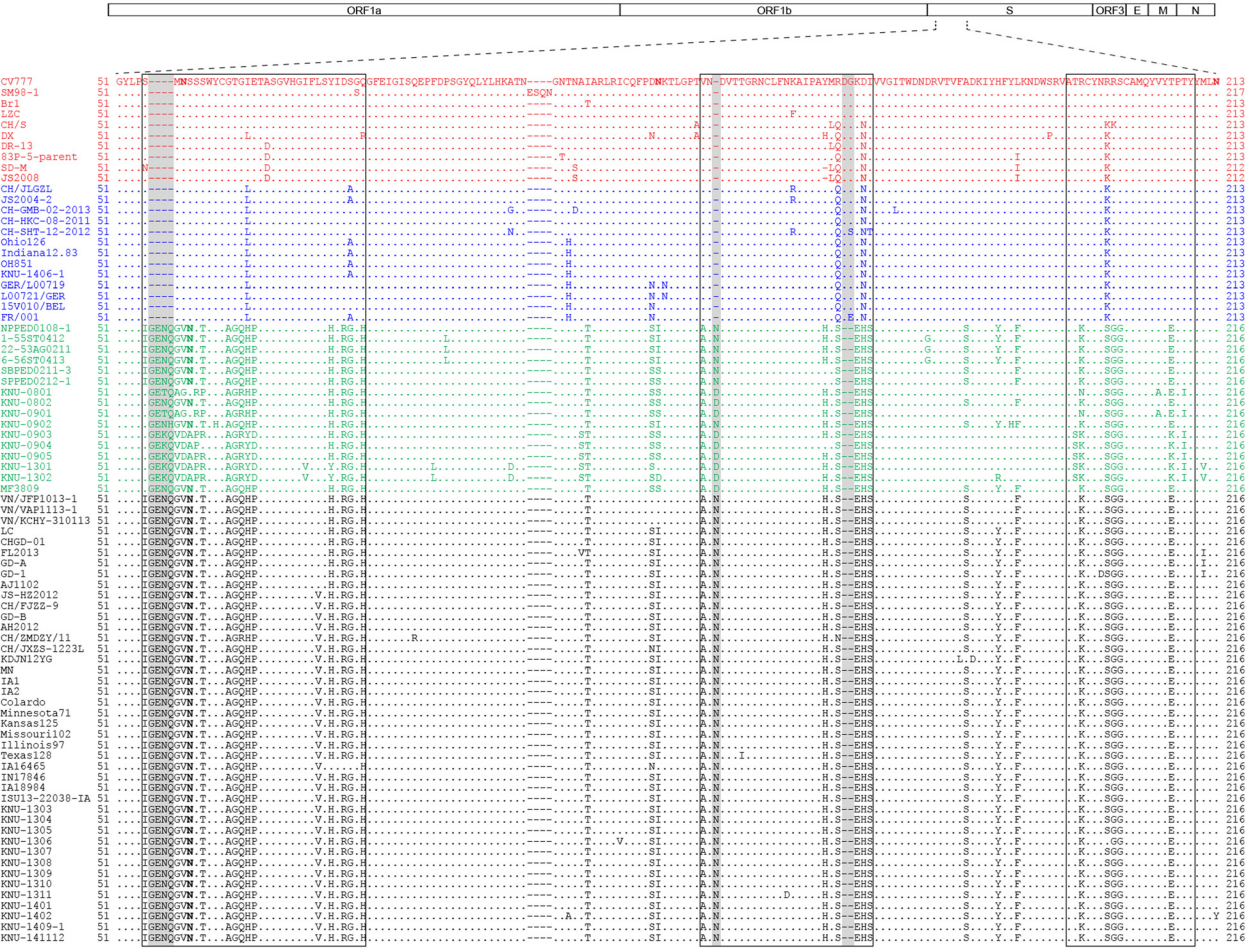
Amino acid sequence alignment of the N-terminal region of the S protein of global PEDV strains. The top illustration represents the organization of the PEDV genome. Only the corresponding alignment of amino acid sequences of the N-terminal region containing hypervariable regions [26] is shown. Dashes (−) indicate deleted sequences. Potential N glycosylation sites predicted by GlycoMod Tool (http://www.expasy.ch/tools/glycomod/) are shown in boldface type. Genetic subgroups of PEDV were marked with different colors: G1a (*red*), G1b (*blue*), G2a (*green*), and G2b (*black*). Insertions and deletions (indels) within PEDV isolates compared to the prototype CV777 strain are shaded. Amino acids representing potential hypervariable domains are indicated by solid boxes

S genes of most PEDV field strains within the G2 group consist of 4161 nucleotides (nt) encoding 1386 amino acid (aa) residues. These genes are 9-nt (3-aa) longer than the homologous gene in the prototype CV777 strain. Compared to the sequences of CV777, G2 PEDV strains possess distinct genetic signatures, S insertions-deletions (S indels) that involve 2 notable 4-aa and 1-aa insertions at positions 55 and 56 and positions 135 and 136, respectively, and a unique 2-aa deletionlocated between positions 160 and 161 within the N-terminal hypervariable region of the S protein [26] (Fig. 4). In addition, this S indel pattern in G2 strains is identical to other novel G2 variants, MF3809 [GenBank:KF779469] and FL2013 [GenBank:KP765609], identified in South Korea and China, respectively, which harbored a large 204-aa S deletion at positions 713–916 or a 7-aa S deletion in the C-terminus, respectively (Fig. 4) [66, 67].

New variant strains within G1b are genetically divergent from G1a and G2 PEDV strains. Although G1b strains were isolated from epidemic cases, they do not contain genetic S signatures typical for G2 field strains. Sequence comparison of the N-terminal one-third of the S gene revealed that G1b strains share more than 95 % of their sequence with G1a classical strains, but their identity with G2 epidemic strains is less than 89 %. In contrast, the analysis of the remaining portion of the S gene indicated that G1b strains exhibit more than 99 % identity with G2 field strains [61]. Furthermore, the entire genome-based phylogenetic analysis showed that G1b strains are clustered closely together with G2 epidemic PEDVs (Fig. 3b). Collectively, these data suggest that novel G1b variants appear to have resulted from a recombination event between classical G1a and epidemic G2 viruses, possibly during viral sg mRNA transcription, which most probably geographically happened in China. The US G1b variants have been named S INDEL strains because of the presence of insertions and a deletion in the S gene compared to sequences of original US PEDV strains [15]. This nomenclature might be incorrect since genomic sequences of PEDV isolates should be initially compared to that of the prototype PEDV strain CV777. Considering this issue, all G2 epidemic isolates include specific S indels, which make them different from CV777. It is therefore recommended that those strains be termed S INDEL strains.

### Molecular epidemiology

#### Epidemiology of PEDV in Europe

Although PEDV first appeared in the United Kingdom and spread to other European countries in the 1970s, the disease impact caused by PEDV in Europe and its economic importance were negligible compared to its effects on the industry in Asian countries and the United States. Over the last decades, therefore, the presence of PEDV was not intensely studied. In 1980s and 1990s, PEDV outbreaks became infrequent, while the virus persisted in an endemic form in the pig population at a low rate. Sporadic outbreaks were reported in some European countries, causing diarrhea in weaner or feeder pigs. A number of serological surveys indicated that seroprevalence of PEDV had become low in European pigs [68–73]. Interestingly, despite low immunity of pigs in European countries, the virus has not been causing severe outbreaks in these susceptible populations although the exact resistance mechanism has not been elucidated. However, PEDV that affected in pigs of all ages, including suckling piglets, re-emerged in a typical epidemic form in Italy in 2006 [74]. In 2014, a case of PED, which occurred on a fattening farm, was reported in Germany [63]. Shortly thereafter, outbreaks of PEDV were identified in a farrow-finish herd in France and in fattening pigs in Belgium [64, 65]. These German, French, and Belgian PEDV strains were found to be genetically almost identical to each other (99.9 % identity) and most closely related to G1b variants identified in China, the United States, and South Korea (Fig. 3). Further surveillance studies are needed to determine whether the G1b strains have already been circulating in Europe or were recently introduced from the United States or Asia. Since PED outbreaks may occur periodically in those countries, the implementation of proper biosecurity protocols would be necessary in order to prevent further spread of PEDV domestically or internationally in Europe. In addition, it would be important to investigate if high virulent G2 PEDV is present in certain areas of Europe.

#### Epidemiology of PEDV in Asia

In Asia, PED epidemics first occurred in 1982 in Japan and since then, PED caused severe epidemics in adjacent Asian countries, particularly in China and South Korea, resulting in heavy losses of piglets [8, 11, 75]. In the late 2000s, PEDV has been reported and become increasingly problematic in the Philippines, Thailand, Taiwan, and Vietnam [10, 17, 76].

In Thailand, several outbreaks of severe PEDV infection have emerged since late 2007. PEDV isolates responsible for epidemics in Thailand had S genetic signatures typical for field epidemic G2 strains and were placed in the cluster adjoining to South Korean and Chinese strains in the G2a or G2b subgroup ([77] see also Fig. 3a). PED was first observed in southern provinces of Vietnam and soon after, the disease spread throughout all major swine-producing regions in that country [76]. Vietnamese strains also had unique S indel characteristics and could be classified as the G2b sublineage, which continues to cause sporadic outbreaks in Vietnam [78].

PED still remains a devastating enteric disease leading to serious losses in China since its first identification. In the early 1990s, a vaccine containing the inactivated prototype CV777 strain was developed and has since been widely used throughout the swine industry in China. Until 2010, outbreaks of PED became infrequent with only a limited number of incidents. However, a remarkable increase in PED epidemics occurred in pig-producing provinces in late 2010 [9]. During that period, new variants of PEDV belonging to the G1b genogroup were first reported in China [9]. In addition, PED outbreaks in vaccinated herds questioned the effectiveness of the CV777-based vaccine [9]. Since then, severe PEDV epidemics have been reported in various regions in China [79–81]. At present, PED outbreaks in China were caused by both G1b variants and field epidemic G2 strains that differed genetically from the prototype CV777 strain [81]. One of the G2b strains, AH2012, was later found to be a potential progenitor of US PEDV strains that emerged subsequently during 2013 [15, 82].

Prior to late 2013, the prevalence of PEDV infection was relatively low with only sporadic outbreaks in Taiwan and Japan. In late 2013, severe large-scale PED epizootics suddenly re-emerged in these countries, which led to tremendous financial losses in their pork industry [17, 18]. Taiwan and Japanese isolates during 2013 to 2014 were phylogenetically related to the same clade as global G2b PEDV strains [17, 18].

#### Epidemiology of PEDV in the United States

PEDV has been exotic in the United States until its sudden and explosive emergence in May 2013. Since then, PEDV has spread rapidly in swine farms across the United States, causing significant financial losses [14]. Genetic and phylogenetic analyses of the emergent US PEDV strains identified during the initial outbreak revealed a close relationship with Chinese strains, especially the AH2012 strain isolated in 2012 from Anhui Province in China, suggesting their origin [82]. Recently, it was suggested that the emergent PEDV strains in the United States potentially descended from 2 Chinese strains, AH2012 [GenBank:KC210145] and CH/ZMDZY/11 [GenBank:KC196276] in G2b sublineage through recombination [15, 80]. Furthermore, PEDV strains similar to those found in the United States appear to be responsible for subsequent large-scale PED outbreaks in South Korea, Taiwan, and Japan in late 2013 [16–18].

In January 2014, other novel US PEDV strains, such as OH851 [GenBank:KJ399978], without typical S protein genetic signatures of the epidemic G2 virus were reported. They phylogenetically clustered closely to novel Chinese strains in the G1b subgroup based on the similarities of the S gene or with the emergent US PEDV strains in the G2 group based on the whole genome characteristics [62]. Novel variants from the United States had a low nucleotide identity in their first 1170 nucleotides of the S1 region and a high similarity in the remaining S gene, compared to the PEDV strains mainly circulating in the United States, suggesting a rapid evolution of US PEDV variants through possible recombination events [62]. However, a retrospective study demonstrated that the new US variants were already present in June 2013, indicating a possibility that multiple parental PEDV strains were introduced into the United States at about the same time [15]. Although another PEDV variant TC-PC22A [GenBank:KM392224] with a 197-aa deletion in the N-terminal region of the S protein was isolated, the large deletion was found to occur during cell adaptation, suggesting that such variants might not circulate naturally in US swine [50].

#### Epidemiology of PEDV in South Korea

The first PED epizootic in South Korea was confirmed in 1992 [8]. However, a retrospective study revealed that PEDV already existed since as early as 1987 [83]. PED outbreaks have since occurred every year and became endemic, which resulted in high rates of death among piglets and substantial economic losses to domestic swine industry until 2010. In a serological survey carried out in 2007, 91.8 % of 159 tested farms had sero-positive pigs in wean to finish periods (30–150 days of age), indicating that the majority of farms were affected with endemic PEDV infection [84]. Since early 2000s, both modified attenuated and inactivated vaccines based on domestic isolates SM98-1 or DR-13 have been introduced nationwide, leading to a decline in the incidence of PEDV-associated diarrheal disease outbreaks compared to the past years. However, continuous PED epidemics in vaccinated farms have raised problems related to the efficacy of Korean commercial vaccines. PED isolates, which prevalently circulated in South Korea during the same period, were classified as G2a strains that contained S indels compared to CV777 and were distantly related to CV777 or Korean vaccine strains belonging to the G1a subgroup [26].

After South Korea experienced severe outbreaks of the foot-and-mouth disease (FMD) in 2010–2011, there was a state of lull during PED emergence. The prevalence of PEDV infections was occasional with only intermittent outbreaks in South Korea from 2011 to early 2013. This epidemic situation likely resulted from the mass culling of more than 3 million pigs (one-third of the entire domestic pig population) in South Korea during the 2010–2011 FMD outbreaks. However, beginning from November 2013, severe PED epidemics increased remarkably and swept through more than 40 % of pig farms across mainland South Korea [16]. Four months later, PEDV hit Jeju Island, which was PEDV-free since 2004 [60]. The re-emergent PEDV isolates responsible for massive epidemics in South Korea in 2013–2014 were classified into the G2b subgroup, where they clustered closely with emergent US PEDV strains [16, 60]. The source of PEDV incursion into the South Korean swine population has not yet been determined. The importation of pig breeding stock during or after the sudden emergence of PEDV in the United States might be one of the possible sources, but it remains unclear whether G2b PEDVs similar to US strains had pre-existed in South Korea. Indeed, the G2b isolates KDJN12YG [GenBank:KJ857475] and KNU-1303 [GenBank:KJ451038] have been identified independently in November 2012 [59] and May 2013 [16], respectively. The former was similar to Chinese G2b strains, whereas the latter resembled emergent US G2b strains (Fig. 3a). Given these results, it is also conceivable that the virus, which has evolved independently by recombination or point mutations might have already been present in South Korea as a minor lineage before the emergence of outbreaks in the United States. Alternatively, it might have originated directly from China. Under suitable circumstances, G2b strains have subsequently become dominant, leading to a number of recent acute outbreaks nationwide [16, 59].

In March of 2014, novel variant G1b PEDV isolates have been found in South Korea, which were similar to the variants reported in China, the United States, and recently in several European countries [61]. They had common genetic and phylogenetic features of G1b strains (no S indels compared to CV777, different phylogenetic subgroup [G1b or G2] depending on the sequence of the S protein or whole-genome, and evidence of recombination), and were most closely related to the US variant strain OH851 among other G1b strains [61]. Although a temporal study will be needed to verify the presence of the G1b virus in earlier periods before its first identification, it is possible that similar to the causes of outbreaks in the United States, 2 G1b and G2b ancestor strains resembling US strains could have been simultaneously transmitted into South Korea. Another novel PEDV G2 strain (MF3809) with a large S deletion was found in South Korea, however, this isolate was identified from only 3 diarrhea samples out of 2634 on 1 out of 569 farms obtained in 2008 [66]. Thus, probability of the existence of this variant in South Korean pigs is very low at present. Nonetheless, we need to continue surveying yet-unidentified PEDV variants that may emerge locally or globally through genetic drift (e.g., after point mutations) or genetic shift (e.g., recombination events).

### Transmission

PEDV infection among pigs occurs principally by a direct or indirect fecal-oral route. Airborne transmission may also play a role in PEDV dissemination under certain conditions [85]. PEDV can mainly enter farms by diarrheal feces or vomitus and contaminated environmental sources via clinically or subclinically infected pigs, trailers (transporting pigs, manures, or food), people (pig owners or visitors, such as swine practitioners or trailer drivers in contaminated work clothing and footwear), or wild animals and birds [6, 86]. Other contaminated fomites, such as sow milk, feed, food items, or food additives or ingredients, including spray-dried porcine plasma, could all be potential sources of the virus [9, 87–89].

After an acute (epidemic) outbreak, PEDV may disappear, remain in the farrowing unit because of inadequate hygiene management (e.g., improper disinfection and poor biosecurity), or persist in pigs in weaning or growing-finishing units where the virus is circulating, causing mild post-weaning diarrhea with very low mortality rates. In this endemic status, if newly born pigs are unable to obtain sufficient levels of maternal immunity from their dams due to incomplete sow vaccination or defective lactation performance owing to mastitis or agalactia, the virus circulating on the farm will infect susceptible piglets, which serve as the source of recurrence of epidemic outbreaks leading to a high number of pig deaths [90, 91]. Such endemic PED circumstances are not restricted to Asia: they may equally happen in North America, where sudden PED epidemics recently emerged.

### Clinical signs, lesions, and pathogenesis

PEDV can infect pigs of all ages, causing watery diarrhea and vomiting accompanied by anorexia and depression. Morbidity approaches 100 % in piglets, but can vary in sows [6]. The incubation period of PEDV is approximately 2 days, ranging from 1 to 8 days depending on field or experimental conditions. The interval between the onset and cessation of clinical signs is 3–4 weeks [4, 6, 49, 81, 92, 93]. Fecal shedding of PEDV can be detected within 48 h and may last for up to 4 weeks. The severity of the disease and mortality rates might be inversely associated with the age of the pigs [6, 94]. PEDV infection in piglets up to 1 week of age causes severe watery diarrhea and vomiting for 3–4 days followed by extensive dehydration and electrolyte imbalance leading to death. Mortality rate averages 50 %, often approaching 100 % in 1- to 3-day-old piglets, and decreases to 10 % thereafter. In older animals, including weaner to finisher pigs, clinical signs are self-limiting within 1 week after the onset of the disease. However, PED may affect growth performance of growing pigs. Sows may not have diarrhea and often they manifest depressive symptoms and anorexia. If farrowing sows lose their offspring, they may subsequently suffer from reproductive disorders including agalactia or delayed estrus, which result from the absence of suckling piglets during the lactation period.

Gross lesions are confined to the gastrointestinal tract and characterized by distended stomach filled with completely undigested milk curd and thin, transparent intestine walls with accumulation of yellowish fluids [49, 92, 95]. Histological hallmarks of the PEDV infection include severe diffuse atrophic enteritis, superficial villous enterocyte swelling with mild cytoplasmic vacuolation, necrosis of scattered enterocytes followed by sloughing, and contraction of the subjacent villous lamina propria containing apoptotic cells [49, 92, 93, 95]. The intestinal villi become reduced to two-thirds or more of their original length (villous height to crypt depth ratios change to less than 3:1 in affected pigs) with the extent of the pathology depending on the stage of the infection or disease process [92, 93, 95].

PEDV replicates in the cytoplasm of villous epithelial cells throughout the small intestine, destroying target enterocytes as a result of massive necrosis or apoptosis. These processes lead to villous atrophy and vacuolation as well as a marked reduction in the enzymatic activity [6, 55]. This sequence of events interrupts digestion and absorption of nutrients and electrolytes, thereby causing malabsorptive watery diarrhea followed by serious and fatal dehydration in piglets [6, 96]. Upon infection with PEDV, the disease outcome and deaths usually occur age-dependently. Although the reasons why PEDV causes more severe disease in nursing piglets in comparison to weaned pigs have not been clearly elucidated, slower regeneration of enterocytes in neonatal pigs may an important factor [97]. PEDV infection increases the number of crypt stem cells and proliferation of crypt cells, pointing to the accelerated epithelial cell renewal [98]. Enterocyte turnover rate was slower in normal nursing piglets than in weaned pigs, suggesting that the speed of crypt stem cell replacement appears to be associated with the age-dependent resistance to PED [98].

### Diagnosis

Since signs of the PEDV infection were clinically and pathologically indistinguishable from those caused by TGEV and the recently described porcine deltacoronavirus [96, 99, 100], PED diagnosis cannot be made purely on the basis of clinical signs and histopathological lesions. Therefore, differential diagnosis to demonstrate the presence of PEDV and/or its antigens must be conducted in the laboratory. A variety of PEDV detection methods, which include immunofluorescence (IF) or immunohistochemistry (IHC) tests, *in situ* hybridization, electron microscopy, virus isolation, enzyme-linked immunosorbent assays (ELISA), and various reverse-transcription polymerase chain reaction (RT-PCR) techniques, have been used. Taking into account their fast turnaround times and sensitivity, conventional and real-time RT-PCR systems available as commercial kits are most widely used for PEDV detection during epidemic or endemic outbreaks, as well as for quarantine or slaughter policies. In addition, nucleotide sequencing of the S gene region may be useful in determining the genotype of PEDV circulating in herds. The combination of RT-PCR and S gene sequencing could well become the optimal tool for monitoring genetic diversity among PEDV isolates.

A number of serological assays have been used for the detection of PEDV antibodies, including indirect fluorescent antibody (IFA) staining, ELISA, and virus neutralization (VN) tests. Due to the special protection strategy (passive immunization) for neonatal piglets against PEDV, determining the presence or absence of anti-PEDV antibodies may be meaningless in sow herds. Instead, measuring quantities (or titers) of neutralizing antibodies against PEDV or, especially, against the S protein in serum and colostrum should be necessary to monitor the immunity level following sow immunization. In this regard, VN test could be essential for estimating levels of protective antibodies, which piglets would receive from sows. However, this method is time-consuming and cannot selectively detect only secretory IgA antibodies representing mucosal immunity. In contrast, IFA and indirect ELISA approaches for antibody detection are equally specific but less time-consuming and easier to perform than VN test. Most assays that are currently in use have been developed on the basis of either whole virus [101–103] or viral protein antigens [29, 104]. Whole virus-based IFA and ELISA tests may be inappropriate for detecting protective antibodies regardless of the antigen (S, M, or N proteins) since they can only detect exposure due to natural infection or vaccination. However, these tools may still be useful for monitoring endemic situation with the PEDV infection in affected farms by determining infection status in weaner to finisher pigs. On the other hand, the entire S protein or its S1 portion could be used as viral antigens for developing ELISA because the S1 domain has been reported to contain the receptor binding region and main neutralizing epitopes [45, 105]. Recently, a recombinant S1 protein-based indirect ELISA has been developed to detect anti-PEDV antibodies [29]. Although this method is a useful, sensitive and specific tool for the detection of anti-PEDV S IgG and IgA antibodies in serum and colostrum samples, it remains to be determined whether concentrations of these antibodies correlate with levels of immune protection.

### Prevention and control measures

#### Biosecurity

One of the most important measures for prevention and control of acute PED outbreaks is strict biosecurity that blocks in principle the entrance of PEDV into pig farms (fattening and farrowing units) by minimizing introduction of any material or any person, which could be in contact with the virus. To accomplish this, disinfection must be thoroughly applied to all fomites, personnel, and external visitors that could be contaminated with PEDV. Although PEDV is inactivated by most virucidal disinfectants [24], PEDV RNA can still be detected by RT-PCR even after disinfection with several commercially available disinfectants [106]. Thus, we may need to evaluate disinfectants in vivo or under various field conditions, especially during the winter season, in order to select suitable disinfectant compositions and appropriate procedures. The following order of disinfection steps is recommended to pork producers attempting to disinfect transportation equipment or affected farrowing units: (i) proper cleaning by a high pressure washer using warm water at temperatures over 70 °C; (ii) disinfection by an appropriate disinfectant according to directions on the label; and (iii) overnight drying [90, 91]. Other biosecurity measures include restricting human traffic between fattening and farrowing units and limiting contact between trailers or drivers and the farm interior during the loading process at the pig farm or between drivers and the slaughter facilities during the unloading process at the collection point [86, 90, 91]. All newly arriving or replacement animals including gilts should be isolated for a certain period to monitor their health status [90, 91].

**Fig. 5:**
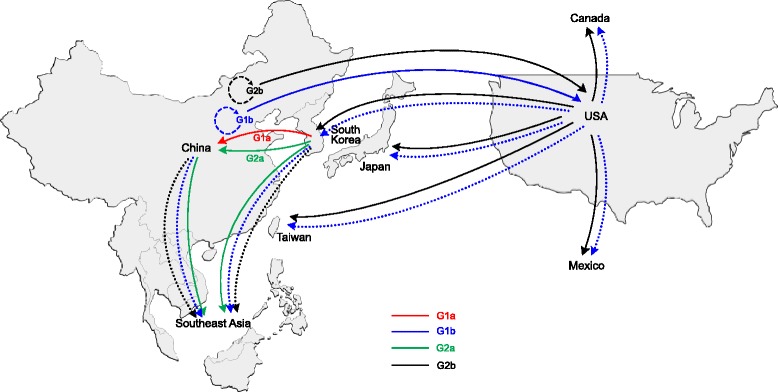
Potential international PEDV transmission routes. Genetic subgroups of PEDV were marked with different colors as described in the legend to Fig. 4. Solid lines indicate PEDV spreads that have already occurred between countries; dotted lines indicate PEDV spreads that are expected to happen eventually; dashed circular arrows denote genetic mutations or recombination events that lead to the emergence of the novel subtypes

PEDV is a transboundary virus that seems to spread readily to neighboring or distant countries even across continents. Since PEDV is not a World Organization for Animal Health reportable disease, quarantine inspection might not properly implement potential sources or routes that mediate virus transmission between countries. During large-scale severe PED epidemics in adjacent or trading countries, quarantine (or international biosecurity) procedures should be adequately reinforced with a particular attention to any risk factors of international disease transmission in order to prevent the entrance of PEDV as well as other emerging or re-emerging pathogens. On the basis of available genetic and phylogenetic data on global PEDV strains, I propose several prospective paths through which PEDV could have spread to Asia and North America (Fig. 5). The classical G1a isolates might have emerged in China due to a careless use of cell-adapted strains as autogenous vaccines or illegal importation of attenuated live vaccines from South Korea. The epidemic G2a South Korean strains might have been initially introduced into China. Chinese G2a viruses were later transmitted to Southeast Asian countries including Thailand and Vietnam. It is also possible that G2a strains in these countries could have been directly transported from South Korea. In China, novel classical G1b and pandemic G2b viruses appear to have arisen concurrently in late 2010 to early 2011 probably via recombination between local G1a and G2a strains or point mutations in resident G2a viruses, respectively. These strains were likely coincidentally introduced into the United States. US-like G1b and G2b strains later landed in South Korea. The genesis of epidemic G2b strains might be ascribed to the evolutionary drift in local G2a lineages in South Korea. In addition, US-like G2b viruses spread further to other North American countries and also to Taiwan and Japan. Currently, there have been no official reports about the emergence of G1b viruses in these countries. Novel G1b and G2b strains may already be present in or they may yet to be brought to Southeast Asian countries from China or South Korea. Considering this expected international dissemination route, we may recheck the importance of quarantine policy against PEDV and other epizootic agents.

#### Vaccines

Vaccination of sows is a fundamental tool in a strategy to control and eradicate PED during epidemic or endemic outbreaks. Piglets are then protected by a transfer of maternal antibodies via colostrum and milk from immune dams. Although PED first emerged in Europe, the disease has not caused sufficient economic losses to justify the vaccine development. In contrast, PED outbreaks in Asian countries have been serious, and therefore several PEDV vaccines have been developed. In China, CV777-attenuated or -inactivated vaccines have been routinely applied against PED. Attenuation of the virulence of the Japanese PEDV strain 83P-5 was achieved after 100 passages in Vero cells [27]. Subsequently, the cell-adapted 83P-5 strain has been employed as an intramuscular (IM) live virus vaccine (P-5 V) in Japan and it is now also available in South Korea. The cell-culture adaptation method was also applied to attenuate two South Korean virulent PEDV strains, SM98-1 (93 passages) and DR-13 (100 passages) [107, 108]. The SM98-1 strain has been used as an IM live or killed vaccine, whereas DR-13 is available as an oral live vaccine. Although these attenuated or inactivated vaccines have been demonstrated to provide protection under experimental conditions, their effectiveness in the field, as well as pros and cons of their use, are still being debated. In South Korea, the multiple dose vaccination program (3 or 4 IM administrations of vaccines in the following order: live-killed-killed or live-live-killed-killed, correspondingly) at 2- or 3-week intervals starting before farrowing or mating is commonly recommended in pregnant sows or gilts to maintain high levels of neutralizing antibodies in serum and colostrum [90, 91]. Recently, the Korean Animal and Plant Quarantine Agency evaluated the efficacy of domestic and imported PED vaccines commercially available in South Korea. The administration of each commercial vaccine according to corresponding manuals (twice at both 3 and 5 weeks prior to farrowing) increased the survival rate of piglets challenged with a virulent wild-type PEDV from 18.2 % to over 80 %. However, all vaccines did not significantly reduce the morbidity rate of diarrhea including virus shedding in feces [109]. Although protection against the enteric disease is primarily dependent on the presence of secretory IgA antibodies in the intestinal mucosa, the vaccine efficacy might be associated with maintaining high levels of PEDV-specific neutralizing antibodies in the serum and colostrum of vaccinated sows [90, 91, 109]. In addition to a direct vaccination, another crucial aspect is passive colostral and lactogenic immunity of neonatal piglets by ample quantities of protective antibodies obtained from sow colostrums and milk. Thus, sanitation and health conditions of lactating sows have to be monitored to eliminate potential factors negatively affecting lactation performance, such as mastitis or agalactia, so that sows could constantly provide high-quality colostrum and milk to their litters. Suckling piglets lose their source of lactogenic immunity at weaning and soon thereafter become vulnerable to PEDV. Following an acute PED epidemic, the virus may persist in susceptible animals or pigs that survived, leading to the circulation of the virus on the farm (endemic PED). Thus, active immunization of weaner to finisher pigs may be necessary for the control of endemic PEDV infections [6].

The low to moderate effectiveness of current PEDV vaccines appears to be due to antigenic, genetic (>10 % amino acid variation between respective S proteins) and phylogenetic (G1 vs. G2) differences between vaccine and field epidemic strains [16, 26, 30, 49, 59]. Therefore, G2b epidemic PEDV or related strains prevalent in the field should be used for the development of next generation vaccines to control PED. The recombinant S1 protein derived from the field G2 PEDV isolate efficiently protected newborn piglets against PEDV, so it could be potentially used as a subunit vaccine for PED prevention in the future [30]. Isolation of PEDV that is phenotypically and genotypically identical to field strains responsible for PED epidemics worldwide is critical for developing effective vaccines. A number of culturable PEDV strains associated with recent outbreaks were obtained in the United States [28, 50]. On the basis of these isolates, an inactivated PEDV vaccine has been developed, which is currently available on the US market. It is expected that commercial live attenuated vaccines will soon be ready for pig producers. In South Korea, a field epidemic PEDV strain has been recently isolated in my laboratory, and we are now utilizing this isolate to spur the development of new effective and safe vaccines [49]. Interestingly, a recent study indicated that previous exposure of sows with “mild” G1b PEDV provides cross-protective lactogenic immunity against piglet challenge with virulent G2b virus [110]. This finding suggests that the vaccine or route of administration may also affect the efficacy of vaccines. Although it may be hard to predict the efficacy of new vaccines in the field, they will be promising practical tools for prevention and/or control of PED if their use is accompanied by tightened biosecurity and optimal farm management.

#### Alternative immunoprophylactic and therapeutic strategies

In acute PED outbreaks with rapidly increasing mortality rates, we may consider intentional exposure (feedback) of pregnant sows to the autogenous virus using watery feces or minced intestines from infected neonatal piglets, which will artificially stimulate rapid lactogenic immunity and, hopefully, shorten the outbreak on the farm [6]. However, there are several complications that need to be considered before the application of this approach. Wide-spread circulation of other viral pathogens, such as PRRSV or PCV2, contained in the intestinal or fecal contents may occur among sows or piglets [111, 112]. Since autogenous viral materials do not have homogenous infectious titers of PEDV, sow immunity may not be induced to a level sufficient for offspring protection. Following artificial exposure of sows, infectious viruses will be shed in feces, which, in turn, could be a potential source for PEDV transmission within the contaminated establishment and between different farms.

Artificial passive immunization by oral administration of specific antibodies represents an attractive approach against gastrointestinal pathogens such as PEDV. Chicken egg yolk immunoglobulin against PEDV or the S1 domain has been found to protect neonatal piglets following challenge exposure [113, 114]. The immunoprophylactic effect of colostrum from cows immunized with PEDV has also led to a reduced mortality in newborn piglets [115]. Single chain variable fragments (scFvs) of the mouse monoclonal antibody or *E. coli* expressing scFvs were verified to neutralize PEDV *in vitro* [116]. This suggests that recombinant *E. coli* harboring scFvs might be an alternative prophylactic measure against PEDV infection. Pharmacological, biological, or natural agents that shorten epithelial cell renewal by stimulating proliferation or reorganization of crypt stem cells could be potential therapeutic targets to reduce PEDV-associated mortality from dehydration following severe villous atrophy [98]. For instance, the epidermal growth factor (EGF) has been shown to stimulate proliferation of intestinal crypt epithelial cells and to mitigate atrophic enteritis induced in piglets by PEDV infection. This finding suggested that treatment with EGF could be another therapeutic option [117]. Broad-spectrum antiviral drugs, such as ribavirin, which suppress PEDV infection *in vitro*, are of interest for their potential to treat PED [53]. Chemical inhibitors as well as compounds from medicinal plants or natural sources, which block the activation of mitochondrial AIF or MAPK signaling pathways required for PEDV replication, could be new therapeutic candidates to reduce PEDV-associated symptoms and mortality [55, 56]. In addition, nutritional supplements, which reduce stress and enhance resistance to the disease, may be useful for PED control in neonatal piglets.

## Conclusions

For the last two or three decades, PED has continued to plague the pork industry in Europe and Asia. In early 2013, the disease struck North America resulting in great economic losses, especially in the United States. Shortly thereafter, massive nationwide PED outbreaks reoccurred in South Korea, Japan, and Taiwan. PED is now globally recognized as an emerging and re-emerging disease and it has become a major financial issue for the swine industry worldwide. Despite geographically limited PED studies in Europe and Asia, a better knowledge of the virology, pathogenesis, immunology, epidemiology, and vaccinology has been gained. Since the emergence of PED in North America, much has been learned from research in the United States. Nevertheless, further studies are warranted to decipher the virus and the associated disease, and more extensive academic and practical studies are needed for a comprehensive understanding of the molecular and pathogenic biology of PEDV in order to develop effective vaccines and establish control measures including biosecurity in affected areas. Lastly, we should bear in mind that a combined application of vaccines, biosecurity protocols, and husbandry management must be the key step to prevent and control PED. Integrated and coordinated efforts in various disciplines among researchers, swine veterinarians, producers, swine industry specialists, producer associations, and authorities are required to achieve effective implementation of necessary measures.
