# Erratum to: Porcine epidemic diarrhea virus: An emerging and re-emerging epizootic swine virus

**DOI:** 10.1186/s12985-016-0465-y

**Published:** 2016-02-01

**Authors:** Changhee Lee

**Affiliations:** Animal Virology Laboratory, School of Life Sciences, BK21 Plus KNU Creative BioResearch Group, Kyungpook National University, Daegu, 41566 Republic of Korea

Erratum

After the publication of this work [1], we noticed that an incorrect version of Fig. 5 (Fig. 1 here) was published. The correct version of Figure five has now been updated in the original article and can also be seen below. The publisher apologises for any inconvenience caused.

**Fig. 5:**
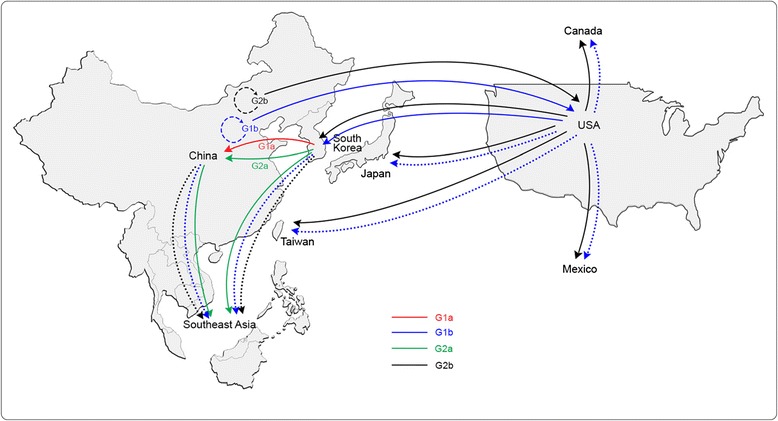
From: Porcine epidemic diarrhea virus: An emerging and re-emerging epizootic swine virus
